# Photosynthesis of CO from CO_2_ with an iron polypyridyl catalyst at a passivated silicon photoelectrode

**DOI:** 10.1039/d5sc05984d

**Published:** 2025-11-05

**Authors:** Gabriella P. Bein, Sergio Fernández, Stephen J. Tereniak, Renato N. Sampaio, Alexander J. M. Miller, Jillian L. Dempsey

**Affiliations:** a Department of Chemistry, University of North Carolina at Chapel Hill Chapel Hill North Carolina 27599-3290 USA dempseyj@email.unc.edu ajmm@email.unc.edu

## Abstract

A first-row transition metal catalyst, [Fe(tpy)(Mebim-py)(NCCH_3_)]^2+^ (tpy = 2,2′:6′,2′′-terpyridine, Mebim-py = 1-methylbenzimidazol-2-ylidene-3-(2′-pyridine)) mediates CO_2_ reduction to CO at passivated p-Si photoelectrodes with applied potentials 240 mV positive of the standard CO_2_/CO reduction potential. The molecular catalyst's selectivity for CO was retained under photoelectrochemical conditions, with negligible direct proton reduction promoted by the photoelectrode. The faradaic efficiency for CO (44 ± 6%) was slightly enhanced relative to the catalyst performance in the dark (33%). A photosynthetic cell based on this photocathode system, coupled with ferrocene oxidation at the anode, successfully operated at a cell voltage of −1.2 V. The photovoltage generated by illumination of p-Si–CH_3_ met and surpassed the potential required for CO_2_ reduction when coupled with ferrocene oxidation. By leveraging a low-overpotential CO_2_ reduction electrocatalyst, a photo-assisted electrochemical efficiency of 0.15% and applied bias photon-to-current efficiency of 0.05% was achieved for this single-junction cell, ultimately storing 46 kJ mol^−1^ (11 kcal mol^−1^) of photon energy.

## Abstract 2

A first-row transition metal catalyst, [Fe(tpy)(Mebim-py)(NCCH_3_)]^2+^, mediates CO_2_ reduction at passivated p-Si photoelectrodes at a cell voltage of –1.2 V, storing 46 kJ mol^−1^ of photon energy in the CO fuel product.

## Graphical abstract

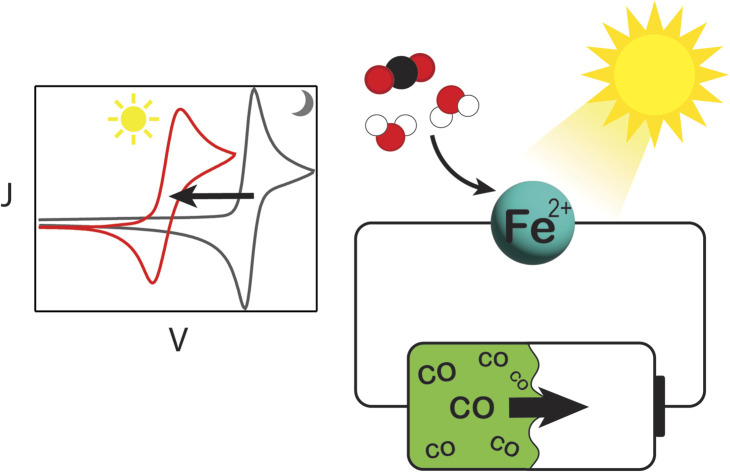

## Introduction

Electricity generation from sunlight using silicon-based photovoltaics has rapidly become a major facet of the global energy portfolio, while efforts to convert photon energy into fuels for long-duration storage or transportation are at a nascent stage.^1,2^ One approach to generating solar fuels is artificial photosynthesis, wherein a light harvester is paired with a catalyst that mediates an energy-storing chemical transformation to produce a fuel. This process ultimately stores the energy of solar photons in chemical bonds, and this energy can be released through combustion or in a fuel cell on-demand. Investing in solar-derived fuels can also improve the circularity of carbon utilization, by upgrading carbon dioxide from a waste material into useful chemicals.^3–5^ In this description, we underscore the distinction between photosynthesis, which is an energy-storing “reverse combustion” reaction, and photocatalysis, which uses light to drive an energy-releasing reaction.^6^

One class of photoelectrochemical cells for artificial photosynthesis integrates molecular catalysts dissolved in the electrolyte solution that are selective for carbon dioxide reduction with visible light absorbing semiconductors.^7–9^ Limitations of this architecture include competition between the CO_2_ reduction reaction at the molecular catalyst and photocathode surface-based redox processes, such as photocathode corrosion and hydrogen evolution.^10–12^ However, passivation of p-type silicon photoelectrodes with polymeric or oxide coatings,^13,14^ or by direct covalent modifications of the Si lattice, has been shown to be effective at improving the photoelectrode stability to surface oxidation and minimizing unwanted side reactivity.^15–18^ These advances, together with the advantages of solution-based catalysts towards well-behaved electrochemical responses, motivate further work in this architecture. Recently, we reported that methyl-terminated p-Si photoelectrodes (p-Si–CH_3_) interfaced with a homogeneous ruthenium polypyridyl catalyst, [Ru(tpy)(Mebim-py)(NCCH_3_)]^2+^ (tpy = 2,2′:6′,2′′-terpyridine, Mebim-py = 1-methylbenzimidazol-2-ylidene-3-(2′-pyridine)), in the electrolyte solution can selectively drive CO_2_ reduction without parasitic hydrogen evolution from the photoelectrode. Unlike unpassivated p-Si–H, which rapidly becomes charge transfer resistant as the surface oxidizes, the p-Si–CH_3_ was stable under photoelectrolysis conditions and sustained a steady photocurrent. The Ru catalyst was able to access the same intrinsic product selectivity for CO at the p-Si–CH_3_ photoelectrode as was observed at metallic electrodes, but with a significant photovoltage of 460 mV.^19^ Importantly, the selectivity for the CO_2_ reduction reaction was rationalized to occur because the reduction of CO_2_ mediated by the ruthenium catalyst kinetically outcompetes direct proton reduction at the photocathode, revealing an important design principle for selective fuel formation.

One disadvantage of the [Ru(tpy)(Mebim-py)(NCCH_3_)]^2+^ system is that this complex has a large overpotential, *η*, (eqn (1)).1
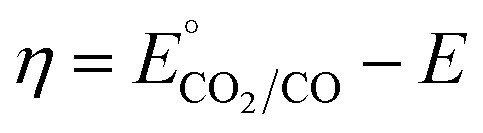


The catalyst requires an applied potential significantly beyond the standard reduction potential for the CO_2_ reduction reaction to form the active catalyst species, with the reduction of the ruthenium species to the active [Ru(tpy)(Mebim-py)]^0^ species occurring at *E*_1/2_ = −1.94 V *vs.* Fc^+/0^.^20^ Under the conditions at which the catalyst operates (95 : 5 CH_3_CN : H_2_O solvent mixture), the standard reduction potential for CO_2_ reduction to CO is −1.44 V *vs.* Fc^+/0^.^21^ Even when paired with an anode reaction that operates with no overpotential, the photovoltage generated at the p-Si–CH_3_ photoelectrode was insufficient to offset an overpotential of this magnitude and a relatively negative applied potential was still needed for photoelectrochemical CO_2_ reduction, such that the system is photocatalytic (Δ*G* < 0), not photosynthetic (Δ*G* > 0).^6^ Furthermore, the full cell potential and efficiency, including both the cathodic and anodic half-reactions, as well as full system losses, must be considered in order to achieve photon energy storage in solar fuels.

We hypothesized that an energy-storing photosynthetic system could be achieved if the same robust silicon photocathode operating with a large photovoltage was paired with a molecular catalyst that operates in the dark with a minimal overpotential. In this case, CO_2_ reduction at the cathode could proceed at an effective “underpotential”, defined as a negative overpotential. In this situation, the applied electrochemical potential is less than the standard reaction potential for the reaction of CO_2_ to CO, and photons supply the remaining thermodynamically required energy. Beyond the impact of demonstrating photon energy storage, an example of energy-storing photosynthesis would provide an opportunity for standardizing efficiency assessment protocols, motiving our work.

**Fig. 1:**
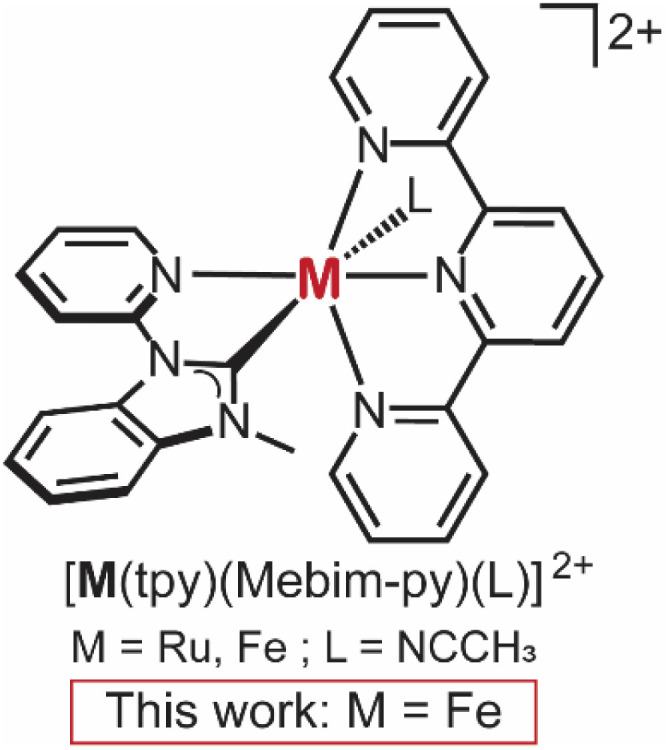
Ru and Fe catalysts compared in this work.

To examine the hypothesis that a low-overpotential catalyst could enable energy-storing photosynthesis in conjunction with a single-junction p-Si photocathode, we identified the [Fe(tpy)(Mebim-py)(NCCH_3_)]^2+^ CO_2_ reduction catalyst (Fig. 1), which mediates CO_2_ reduction to CO by a similar mechanism to the analogous Ru complex, but operates at a substantially reduced overpotential for CO_2_ reduction (*η* = 150 mV).^22^ Combining this low-overpotential catalyst based on an abundant first-row transition metal element with p-Si–CH_3_, a durable and well-passivated visible-light-absorbing photocathode based on the industry standard photovoltaic material, we access a photocathode system competent for CO_2_ reduction with faradaic efficiencies (FEs) exceeding those achieved with a metallic electrode. Pairing this photocathode with ferrocene oxidation at a dark anode affords a photosynthetic system competent for CO_2_ reduction. While many works focus solely on the CO_2_ reduction reaction that produces the fuel of interest, it is important to carefully consider both half-reactions when designing a photosynthetic cell in order to establish energy-storing properties. Operating at an applied cell potential of −1.2 V under 1 sun illumination, 46 kJ mol^−1^ (11 kcal mol^−1^) of photon energy are stored in the CO fuel product. This demonstration is one of the first known examples of true energy-storing photosynthesis based on a molecular catalyst operating with a single-junction semiconductor and provides a blueprint for advancing photon-to-fuel efficiency in molecular catalyst-based photoelectrosynthesis systems.

## Results and discussion

### Cyclic voltammetry of [Fe(tpy)(Mebim-py)(L)](PF_6_)_2_ at p-Si–CH_3_

**Fig. 2:**
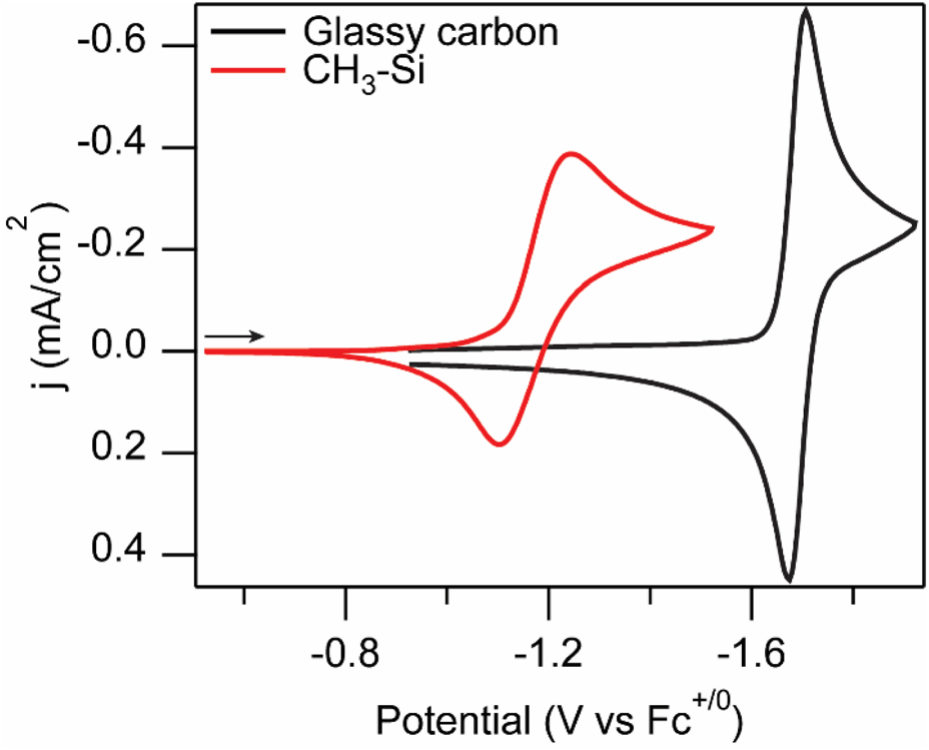
Cyclic voltammograms of 1 mM [Fe(tpy)(Mebim-py)(NCCH_3_)][PF_6_]_2_ solution at an illuminated p-Si–CH_3_ (red), and glassy carbon electrode (black). Voltammograms recorded at 100 mV s^−1^ in 100 mM [NBu_4_][PF_6_] 95 : 5 CH_3_CN : H_2_O solution under N_2_ atmosphere with a Pt mesh counter electrode, and an Ag/AgNO_3_ reference electrode. Light source was a warm white light LED with irradiance of 339 mW cm^−2^ (power measured for *λ* = 439 nm). Arrow indicates scan direction.

The p-type CH_3_-terminated Si photoelectrodes (p-Si–CH_3_) were prepared according to a published procedure.^19^ Cyclic voltammograms of [Fe(tpy)(Mebim-py)(NCCH_3_)]^2+^, synthesized as previously reported,^22^ were recorded with a glassy carbon working electrode in the dark and with a p-Si–CH_3_ photoelectrode under illumination (Fig. 2). At glassy carbon, [Fe(tpy)(Mebim-py)(NCCH_3_)]^2+^ undergoes a two-electron reduction with a half-wave potential, *E*_1/2_ = −1.69 V *vs.* Fc^+/0^ (peak-to-peak separation, Δ*E*_p_ = 0.032 V), consistent with the previous report.^22^ At p-Si-CH_3_ under illumination, *E*_1/2_ = −1.22 V *vs.* Fc^+/0^ (Δ*E*_p_ = 0.14 V) indicating a photovoltage of *ca.* 470 mV. This photovoltage is consistent with those observed previously in the related [Ru(tpy)(Mebim-py)(NCCH_3_)]^2+^ system, where the photovoltage quantified by the shift in *E*_1/2_ was equivalent to the value determined through other methods.^19^ The voltammogram recorded at glassy carbon has a smaller peak-to-peak separation and narrower full width at half maximum, but passes a similar amount of charge as that of p-Si–CH_3_. We note that the [Fe(tpy)(Mebim-py)(NCCH_3_)]^2+^ solution absorbs in the visible light spectrum but the molar absorption coefficient (1392 M^−1^ cm^−1^ at 507 nm, Fig. S1) is lower than that of the Ru catalyst.^19^

**Fig. 3:**
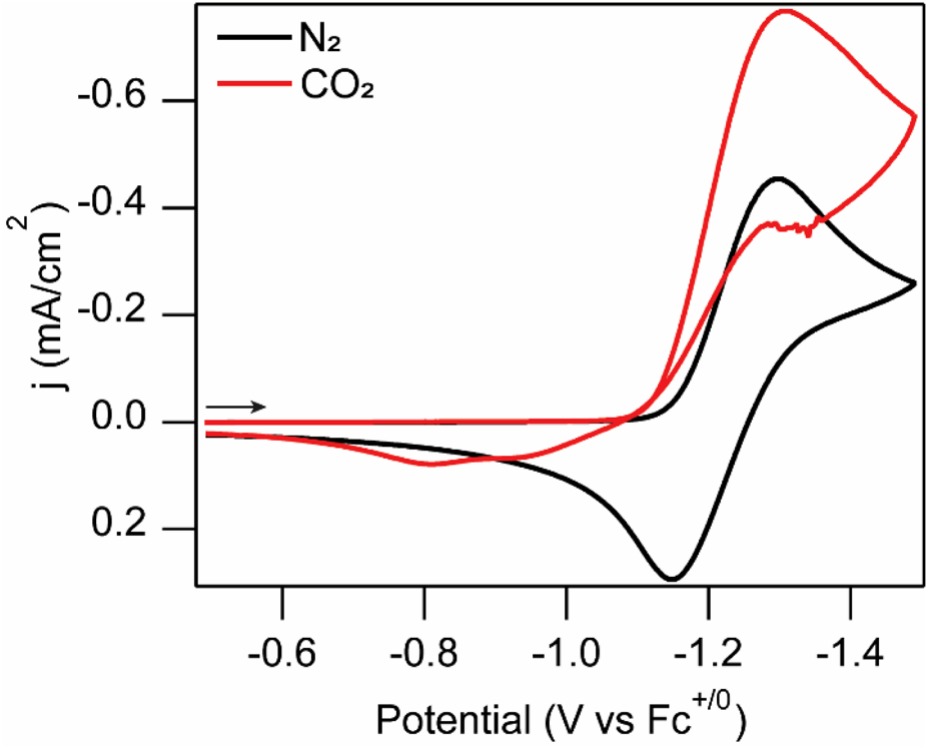
Cyclic voltammograms of 1 mM [Fe(tpy)(Mebim-py)(NCCH_3_)][PF_6_]_2_ solution at an illuminated p-Si–CH_3_ photoelectrode under a N_2_ (black) and CO_2_ atmosphere (red). Voltammograms recorded at 100 mV s^−1^ in 100 mM [NBu_4_][PF_6_] 95 : 5 CH_3_CN : H_2_O solution with a Pt mesh counter electrode (in a separate compartment), and an Ag/AgNO_3_ reference electrode. Light source was a warm white light LED with an irradiance of 339 mW cm^−2^. Arrow indicates starting point and scan direction.

Under a CO_2_ atmosphere, we observe loss of chemical reversibility of the Fe^2+/0^ wave and an enhancement of the cathodic current at the p-Si–CH_3_ (Fig. 3). This peak-shaped wave is interpreted as a four-electron, two-proton reduction of the Fe^II^ species coupled to CO_2_ reduction to form the CO-bound Fe^0^ species, Fe(tpy)(Mebim-py)(CO), based on reactivity observed in the dark at metallic electrodes.^22^ CO release enables catalyst turnover, but the slow kinetics for this catalyst (TOF = 8 s^−1^) manifest in minimal current enhancement on the cyclic voltammetry timescale. A new oxidation feature at −0.80 V *vs.* Fc^+/0^ is apparent in the return sweep, heralding the formation of Fe(tpy)(Mebim-py)(CO), which has a characteristic oxidation at this potential.^22^ These voltammograms indicate that the Fe catalyst is sufficiently photostable under these conditions and can mediate CO_2_ reduction at very mild applied potentials with a p-Si–CH_3_ photoelectrode under illumination, notable since the photocatalytic activity is observed at potentials more positive than the standard electrode potential for CO_2_ to CO in the dark (
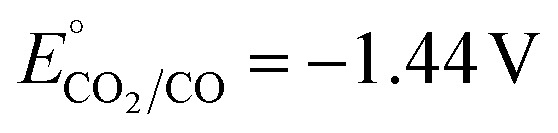
*vs.* Fc^+/0^ in 95 : 5 CH_3_CN : H_2_O solvent mixture).^21^ There is a trade-off between the catalyst overpotential and its activity, as seen in how the peak catalytic photocurrent (*i*_p,c_) for [Fe(tpy)(Mebim-py)(NCCH_3_)]^2+^ at 0.1 V s^−1^ is substantially lower than that recorded for [Ru(tpy)(Mebim-py)(NCCH_3_)]^2+^ under the same conditions (Fig. S2). This observation is consistent with the relative current response of these two catalysts observed at glassy carbon electrodes,^22,23^ which is in line with the three order-of-magnitude difference in their respective turnover frequencies. It is significant that the photoelectrochemical response at p-Si–CH_3_ is well-behaved and closely resembles the voltametric waveforms at glassy carbon; the well-defined and interpretable cyclic voltammograms contrast the commonly observed broad and low current amplitude redox events of p-Si photoelectrodes modified with molecular catalysts on the surface.^24–27^ Furthermore, the comparable photovoltages achieved for the Fe and Ru systems exceeded expectations, as p-Si photovoltage is known to correlate with *E*°′ of the redox-active analyte in solution until inversion conditions are reached.^28^

### Photoelectrocatalytic CO_2_ reduction in a three-electrode cell configuration

**Fig. 4:**
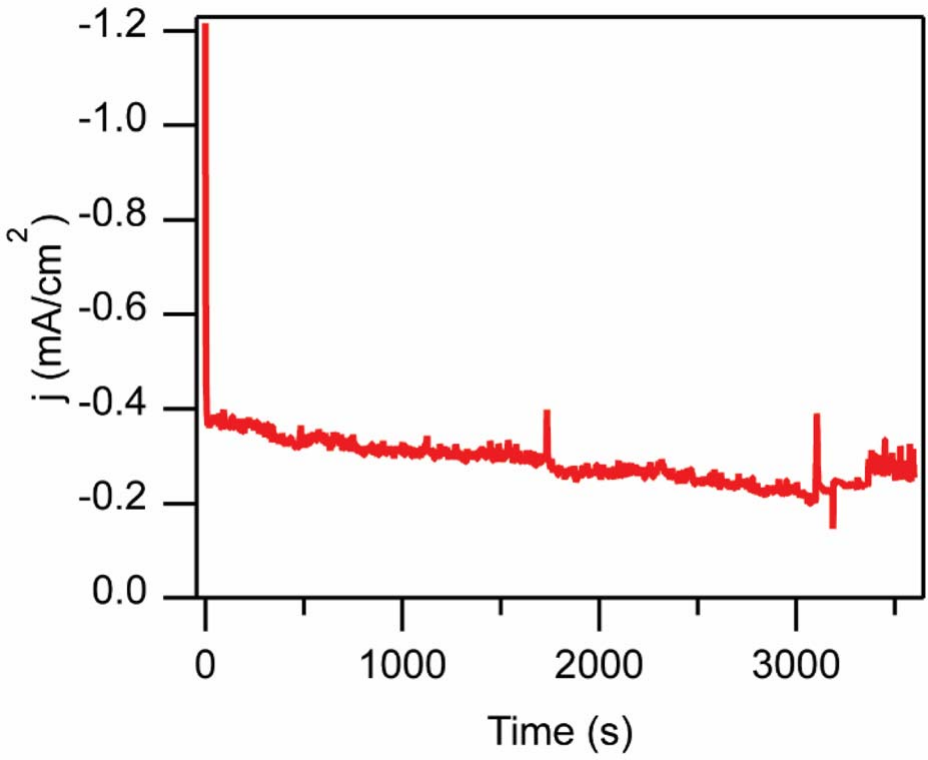
Controlled potential photoelectrolysis (CPPE) of the stirred solution at *E*_app_ = −1.31 V *vs.* Fc^+/0^ over 3600 s. CPPE recorded in divided electrolysis cell at 100 mV s^−1^ in 100 mM [NBu_4_][PF_6_] 95 : 5 CH_3_CN : H_2_O solution with a p-Si–CH_3_ photoelectrode, Pt mesh counter electrode (in separate compartment), and 10 mM Ag/AgNO_3_ reference electrode with AM1.5G illumination (100 mW cm^−2^).

Controlled potential photoelectrolysis (CPPE) experiments in an H-cell configuration (Fig. S3) were employed to evaluate the p-Si–CH_3_|[Fe(tpy)(Mebim-py)(NCCH_3_)]^2+^ system for catalytic CO_2_ reduction. An applied potential of −1.31 V *vs.* Fc^+/0^ was selected for these experiments based on the peak potential of the catalytic wave seen in the cyclic voltammogram. This applied potential is 130 mV more positive than the CO_2_/CO thermodynamic reduction potential, and 90 mV more negative of the *E*_1/2_ of Fe^2+/0^ couple recorded at the same p-Si–CH_3_ photoelectrode. After an initial spike, the photocurrent density decreased moderately over the course of the 3600 s CPPE, from −0.40 to −0.25 mA cm^−2^ (Fig. 4).

After a 3600 s CPPE, during which −0.374 C was passed, the headspace of the reaction vessel was analysed by gas chromatography for gaseous product detection. CO was detected with a FE of 52% and no H_2_ was detected. The FE_CO_ value is slightly higher than that reported for this catalyst at glassy carbon electrodes under static headspace conditions in the dark (33%). Based on mechanistic investigations in previous studies of this catalyst, we attribute the remainder of the charge passed to the build-up of the Fe(tpy)(Mebim-py)(CO) intermediate in solution.^22^ Voltammograms recorded immediately before and after CPPE are shown in Fig. S4. The p-Si–CH_3_ photoelectrode was durable under the photoelectrocatalytic conditions, evidenced by the lack of competitive HER activity and by the sustained ability to pass charge, as observed previously.^19^ With AM1.5G illumination at −1.31 V *vs.* Fc^+/0^, the average across three trials under the same conditions were FE_CO_ = 44 ± 6% and FE_H_2__ = 1 ± 1%. The product selectivity was also reproducible at various applied potentials and with different illumination sources, with FE_CO_ ranging from 33–76%, depending on the conditions (Table S1).

### Photoelectrosynthesis of CO in a two-electrode cell

After observing high selectivity for CO_2_ photoelectrolysis at the peak potential of the catalytic wave, we sought to construct a photosynthetic cell to quantify how much energy could be stored in the solar fuel. The overpotentials for the redox half-reactions at the anode and the cathode affect the overall energy efficiency of the photosynthetic process. Improving energy efficiency is critical to increasing the viability of fuels synthesized through artificial photosynthesis as a suitable alternative to fossil fuels.^29^

Traditionally, CO_2_ reduction electrolysers have coupled the reductive half-reaction with oxygen evolution from water (OER) at the anode.^30^ However, depending on the catalyst employed, water oxidation often operates with a high overpotential because of slow reaction kinetics,^31,32^ consuming up to 90% of the total cell voltage.^33^ In some reports, OER is replaced with organic alcohol oxidation at the anode to in order to operate the photosynthesis cell at moderate cell voltages and improve energy conversion efficiency.^33–37^ Here, we selected ferrocene oxidation as a convenient and well-behaved anode half-reaction with a well-defined potential (*E*_1/2_ (Fc^+/0^) = 0 V *vs.* Fc^+/0^) that can operate at essentially zero overpotential, leading to the overall reaction of eqn (2) for our proof-of-concept demonstration. A two-electrode CPPE was performed with 1 mM [Fe(tpy)(Mebim-py)(NCCH_3_)]^2+^ in the working compartment and 10 mM Fc and 10 mM [Fc][PF_6_] in the counter electrode compartment. The 1 : 1 Fc : Fc^+^ was chosen for the counter compartment to approach equilibrium conditions for eqn (2) and to aid in measuring the change in open circuit potential (OCP) of the counter compartment during the photoelectrolysis. The cell resistance measured between the cathode and anode across the dividing glass frit of the H-cell was ca. 1 kΩ, showing significant potential drop as a result of the photosynthetic cell geometry.2
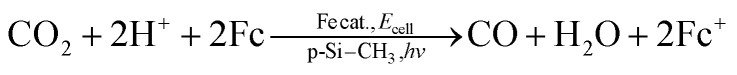


**Fig. 5:**
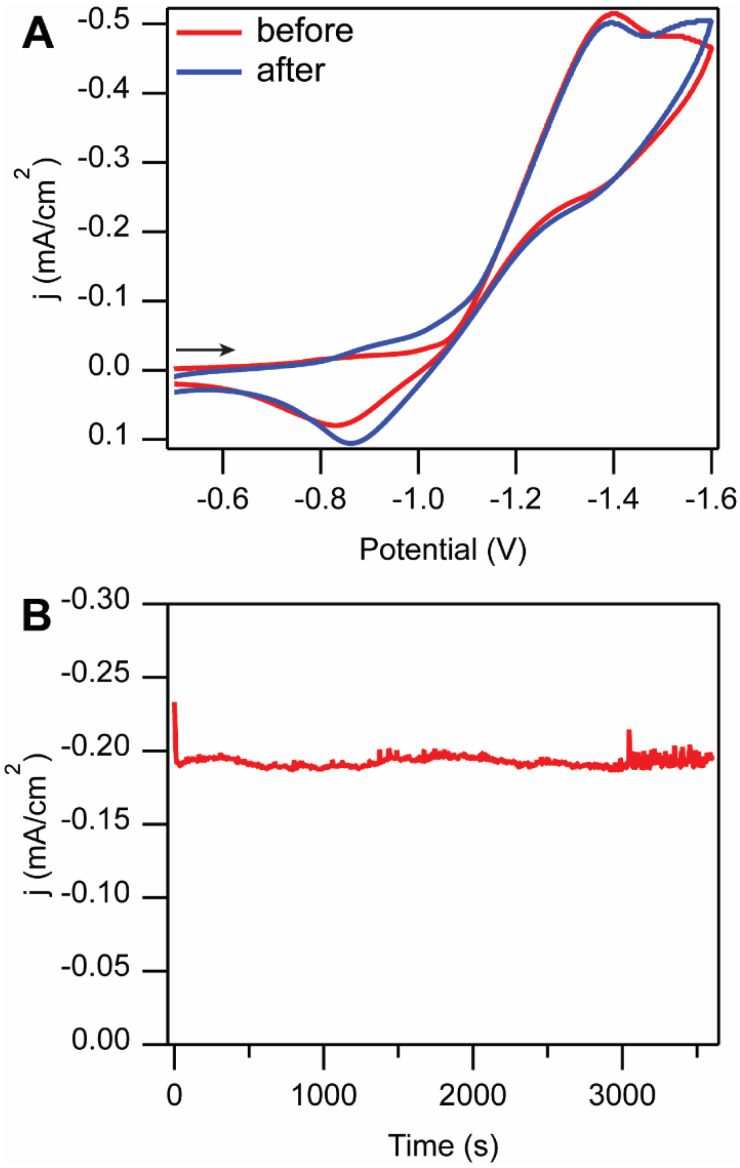
(A) Cyclic voltammograms of 1 mM [Fe(tpy)(Mebim-py)(NCCH_3_)]^2+^ catalyst solution under CO_2_ atmosphere before (red) and after (blue) photoelectrolysis. (B) Controlled potential photoelectrolysis of stirred solution at *E*_cell_ = −1.2 V over 1 h. Voltammograms and CPPE recorded in divided electrolysis cell at 100 mV s^−1^ in 100 mM [NBu_4_][PF_6_] 95 : 5 CH_3_CN : H_2_O solution with p-Si–CH_3_ photoelectrode, Pt mesh counter electrode (in separate compartment), and 10 mM Ag/AgNO_3_ reference electrode with AM1.5G illumination (100 mW cm^−2^). Arrow indicates starting point and scan direction.

Under AM1.5G 100 mW cm^−2^ simulated solar illumination, a cell potential (*E*_cell_) of −1.2 V was applied for 1 h between the p-Si–CH_3_ photocathode and the Pt mesh anode (Fig. 5). Under illumination, this cell voltage was sufficient to drive the reaction forward, passing on average 0.276 C of charge and producing CO with 56 ± 18% FE (Table S2). The OCP of the counter compartment shifted to more positive potentials, consistent with the oxidation of ferrocene to ferrocenium. The production of Fc^+^ at Pt mesh had near unity FE (96 ± 2%, see Table S3).

The Gibbs free energy (Δ*G*) of the net cell reaction defined in eqn (2) can be used to quantify how much light energy is stored in the fuel:3
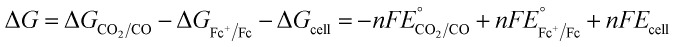
where *n* is the number of electrons in the reaction (2) and *F* is Faraday's constant (96 485 C mol^−1^). The applied voltage *E*_cell_ is 240 mV less negative than the potential associated with the CO-producing reaction of eqn (2) at the cathode (
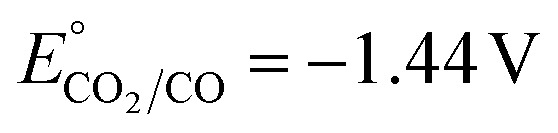
*vs.* Fc^+/0^ in 95 : 5 CH_3_CN : H_2_O solvent mixture) with ferrocene oxidation at the anode.^21^

With *E*_cell_ = −1.2 V, we find Δ*G* = 46 kJ mol^−1^ (11 kcal mol^−1^). As the reaction is endergonic under state conditions, the photosynthetic cell requires the incident light energy to drive this reaction, ultimately storing 46 kJ mol^−1^ of photon energy in the CO fuel product.^6^

The photo-assisted electrochemical efficiency (*η*_PAE_) of this photosynthetic cell is the ratio of the system output to total input power:^38,39^4
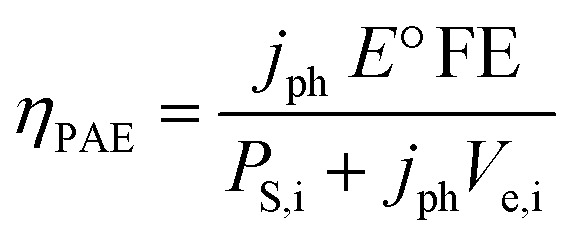
in which *j*_ph_ is the photocurrent density at the potential under evaluation (0.192 mA cm^−2^), *P*_S,i_ is the input power from illumination (100 mW cm^−2^), and *V*_e,i_ represents the input voltage required to drive photoelectrolysis at the operating photocurrent. In a two-electrode cell configuration, *V*_e,i_ = *E*_cell_.

An overall *η*_PAE_ of 0.15% is quantified for this photosynthetic cell operating at −1.2 V. In context, the *η*_PAE_ quantified here is on the low end compared to heterogeneous photoelectrochemical CO_2_ reduction systems. While *η*_PAE_ is rarely quantified in photoelectrochemical CO_2_ reduction studies, there are reports for heterogeneous systems with *η*_PAE_ ranging from 0.37% to 11%.^40–43^ To the best of our knowledge, there are no other reports to date of *η*_PAE_ for a molecular catalyst driving photoelectrochemical CO_2_ reduction catalysis.

With this data we can also calculate the related applied bias photon-to-current efficiency (ABPE) metric. By nature of utilizing a single-junction p-Si photoelectrode, an applied bias is required to supplement the photovoltage and drive CO production. ABPE represents the net chemical power output relative to the incident solar power:^38,39,44^5
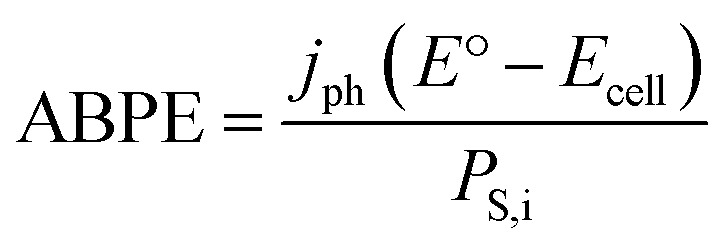


Applying eqn (5), the ABPE is 0.05%. This metric is commensurate with work by Li and co-workers, who reported a similar ABPE of 0.06% for a cobalt polypyridyl catalyst attached to carbon nanotubes on a TiO_2_-coated Si photoelectrode.^45^ While some photo-assisted electrochemical efficiency loss comes from the FE of the cathodic reaction (which can be improved by operating under constant CO_2_ flow),^22^ the major contributing factor to both low *η*_PAE_ and low ABPE is likely the low photocurrent, which we hypothesize is limited by low catalyst turnover frequency and by low internal quantum efficiency (IQE) of the photocathode. Slow catalyst kinetics lead to efficiency-limiting carrier recombination when the catalyst is not available to accept photogenerated charge carriers. A faster catalyst could increase the photon-to-current efficiency by keeping pace with the incident photon flux. The IQE is affected by charge separation, charge carrier recombination, and the heterogeneous electron transfer rate.^19,46^ Indeed, previous studies of the [Ru(tpy)(Mebim-py)(NCCH_3_)]^2+^-mediated CO_2_ reduction at p-Si–CH_3_ showed that photocurrent was limited by the IQE.^19^ IQE can be improved by increasing electron-transfer rate constants and with the use of high-quality Si wafers. These mechanisms for increasing *j*_ph_ provide a roadmap towards improving the efficiency metrics, *η*_PAE_ and ABPE. Further, despite the low *η*_PAE_ and ABPE values, this work demonstrates exciting proof-of-concept for storing incident photon energy as fuel in photosynthetic system with a molecular catalyst-based photocathode operating with a single-junction semiconductor light harvester.

## Conclusions

In conclusion, we have demonstrated one of the first examples of a photosynthetic cell based on a molecular catalyst and a single-junction photocathode competent for CO_2_ reduction. The photovoltage generated by the p-Si–CH_3_ photoelectrode exceeded the overpotential required for the Fe catalyst, allowing for operation at a cell voltage representing an “underpotential” 240 mV more positive than the standard reduction potential for CO_2_. Ultimately, this system stored 46 kJ mol^−1^ (11 kcal mol^−1^) of photon energy in the CO product, with slightly enhanced FE under photoelectrochemical conditions compared to metallic electrodes. An overall photo-assisted electrochemical efficiency of 0.15% and an applied bias photon-to-current efficiency of 0.05% was quantified, which provides valuable benchmarking information on the solar-to-fuel energy conversion and on the potential for future improvements for the field. This molecular photoelectrosynthetic system represents an important step forward in solar energy storage towards renewable fuels.

## Author contributions

G. P. B., S. F., J. L. D, and A. J. M. M. conceptualized the project. J. L. D. and A. J. M. M. supervised the work. G. P. B. prepared the Si electrodes and photoelectrodes. S. F. and S. J. T. synthesized the catalysts. G. P. B. and S. F. performed the photoelectrochemical experiments. G. P. B, S. F., R. N. S., A. J. M. M., and J. L. D. contributed to experiment design and data analysis. G. P. B. and J. L. D. wrote the original manuscript draft. All authors contributed to discussions on the data and to the development of the manuscript.

## Conflicts of interest

There are no conflicts to declare.

## Supplementary Material

SC-OLF-D5SC05984D-s001

## Data Availability

The data supporting this article have been included as part of the supplementary information (SI). Supplementary information: experimental methods, absorption spectra, cyclic voltammetry, faradaic efficiency calculations. See DOI: https://doi.org/10.1039/d5sc05984d.
