# Supplementary material for: Photosynthesis of CO from CO2 with an iron polypyridyl catalyst at a passivated silicon photoelectrode
Source: Chem Sci. 2025 Nov 5;16(48):23005–11. doi: 10.1039/d5sc05984d (PMC12587449; doi:10.1039/d5sc05984d)
Supplement: SC-016-D5SC05984D-s001 [file SC-016-D5SC05984D-s001.pdf]

Supplementary Information (SI) for Chemical Science.
This journal is © The Royal Society of Chemistry 2025

Supporting Information

Photosynthesis of CO from CO_2 with an Iron Polypyridyl Catalyst at a Passivated Silicon Photoelectrode

Gabriella P. Bein, Sergio Fernández, Stephen J. Tereniak, Renato N. Sampaio, Alexander J. M. Miller,* Jillian L. Dempsey*

Department of Chemistry, University of North Carolina at Chapel Hill, Chapel Hill, North Carolina 27599-3290, United States

Experimental Methods

General Considerations. 380 ± 25 μm thick, p-type (boron doped, 1–20 Ω cm^{-1}), and 380 ± 25 μm thick, n^+-type (arsenic doped, 0.001–0.005 Ω cm^{-1}), single-side polished CZ silicon (111) wafers were acquired from NOVA electronic materials. Tetrabutylammonium hexafluorophosphate (TCI, >98%) was recrystallized twice from hot ethanol, washed with cold ethanol, and dried under vacuum. Acetonitrile (Fischer Chemical, HPLC grade) was dried using a solvent system from Pure Process Technology. All other materials were commercially available and used as received, unless otherwise noted. UV-Visible absorbance spectra were recorded on an Agilent Technologies Cary 8454 UV-Vis spectrophotometer with a tungsten lamp within a quartz cuvette. $[Fe(tpy)(Mebim\text{-}py)(NCCH_3)][PF_6]_2$[1] and $[Ru(tpy)(Mebim\text{-}py)(NCCH_3)][PF_6]_2$[2] were synthesized according to reported literature procedures.

UV-Vis Absorbance Characterization of $[Fe(tpy)(Mebim\text{-}py)(NCCH_3)][PF_6]_2$. UV-Vis absorbance spectra of $[Fe(tpy)(Mebim\text{-}py)(NCCH_3)](PF_6)_2$ samples ranging in concentration from 5.8×10^{-5} to 2.2×10^{-4} M in CH_3CN were collected from 190–1100 nm with a 1 cm pathlength and background corrected for solvent absorption. A Beer-Lambert plot was constructed for absorption at 507 nm and $\varepsilon = 1300\ M^{-1}\ cm^{-1}$ was extracted from the slope of the linear regression ($R^2 = 0.9938$).

Silicon Photoelectrode Preparation. Low doped p-type CH_3-terminated Si (111) wafers (p-Si–CH_3) were prepared according to a literature procedure.[3] The p-Si–CH_3 wafers were fashioned into ca. 0.5 cm^2 photoelectrodes, as described previously.[2] The precise surface area was measured by photographing each photoelectrode and analyzing the image dimensions with the program ImageJ.

Cyclic Voltammetry. Cyclic voltammograms of [Fe(tpy)(Mebim-py)($NCCH_3$)][PF_6]$_2$ and [Ru(tpy)(Mebim-py)($NCCH_3$)][PF_6]$_2$ were recorded using a Pine Research WaveDriver 40 DC Bipotentiostat/Galvanostat. Measurements were conducted in a glass scintillation vial containing a 3 mm glassy carbon disc working electrode or the p-Si–CH_3 photoelectrode. Solutions contained 1 mM of the analyte in 100 mM [NBu_4][PF_6] 95:5 CH_3CN:H_2O supporting electrolyte solution. Platinum gauze, 50 x 50 mm made of 52 mesh woven from 0.1 mm wire (Fischer Scientific), was used as the counter electrode. Ag wire in a fritted capillary containing the same electrolyte solution served as a pseudoreference electrode. The cell was sparged for 20 minutes with Ar or CO_2. Illumination source to p-Si–CH_3 was a ThorLabs MWWHL3 warm white light source of irradiance 339 mW/cm^2 (power measured for λ = 439 nm at an optical distance of 0.5 cm), through the glass sidewall of the scintillation vial. Unless otherwise specified, voltammograms were collected at 100 mV/s and externally referenced to a 10 mM ferrocene/ferrocenium redox couple with a 3 mm glassy carbon disc working electrode.

Three Electrode Controlled Potential Photoelectrolysis. Electrolyte solutions containing analytes of interest for bulk electrolysis were prepared in a nitrogen glovebox and placed in a custom air-tight electrochemical borosilicate H-cell with a glass frit dividing working and counter compartments. The working compartment included the p-Si–CH_3 photoelectrode and 10 mM Ag/$AgNO_3$ reference electrode in a 1 mM [Fe(tpy)(Mebim-py)($NCCH_3$)][PF_6]$_2$ and 100 mM [NBu_4][PF_6] supporting electrolyte solution prepared in 95:5 CH_3CN:H_2O. The potential was externally referenced with a 3 mm diameter glassy carbon disc working electrode to the $Fc^{+/0}$ couple at 0 V at the start and end of the experiment. The counter compartment included a 50 x 50 mm platinum 52 mesh counter electrode in the supporting electrolyte solution. The cell was sealed, and the solution sparged with CO_2 for 20 min. The gas purge ports were covered with septa after

the stopcocks were closed. A gas bridge connected the headspace of working and counter compartments to equilibrate pressure. Controlled potential electrolysis was performed with a CH Instruments 700E potentiostat for 1 hour with an 8 x 3 mm Teflon-coated stir bar stirring at ~1000 rpm in each compartment. Illumination was either 1) a Newport Oriel Class ABA Solar Simulator (Model 91191-1000) calibrated to 1 Sun (100 mW/cm^2) illumination using a Newport Reference Cell and Meter (Model 91150V), operating with a 400 nm long pass filter or 2) a ThorLabs MWWHL3 warm white LED of (WWLED) at 339 mW/cm^2 (power measured for λ = 439 nm at an optical distance of 0.5 cm) calibrated to a Newport power meter (Model 843-R-USB) coupled with a Newport Thermopile Sensor (Model 919P-010-16) for measuring broadband light sources. All illumination is quantified as incident irradiance, illumination occurred through the curved, borosilicate sidewall of the H-cell. Illuminations source is denoted in every figure or table.

Two-electrode Controlled Potential Photoelectrolysis. Bulk photoelectrolysis was set up as described above, but with 10 mM ferrocene and 10 mM ferrocenium hexafluorophosphate added to the electrolyte solution of the anode compartment. The electrodes were a ca. 0.5 cm^2 p-Si–CH_3 photocathode and a 50 x 50 mm platinum 52 mesh anode. The open current potential of the anode solution before and after photoelectrolysis was measured against an Ag/$AgNO_3$ reference using the same potentiostat and confirmed by voltage measurement with a multimeter. A two-electrode controlled potential electrolysis experiment was performed with a CH Instruments 700E potentiostat for 1 hour with the cell potential at –1.2 V. Solar simulator illumination was provided with a Newport 1 kW Xenon Arc Lamp with Air Mass 1.5G (AM1.5G) filter at 100 mW/cm^2 with a 400 nm long pass filter as described above.

Gas Chromatography. Following controlled potential photoelectrolysis experiments, a 500 µL headspace sample was extracted from the H-cell working compartment with a Pressure-Lok®

Precision Analytical Syringe (Vici Precision Sampling). The sample was analyzed using an Agilent Gas Chromatograph 8890 instrument with Ar carrier gas. CO was quantified by integration of the methanizer-equipped flame ionization detector peak that eluted at ca. 7 minutes relative to a calibration curve of known CO concentrations. H_2 was quantified by integration of the thermal conductivity detector peak that eluted at ca. 2.4 minutes relative to a calibration curve of known H_2 concentrations. Product peaks that were not integrable (<1 a.u.) were considered not detected (n.d.).

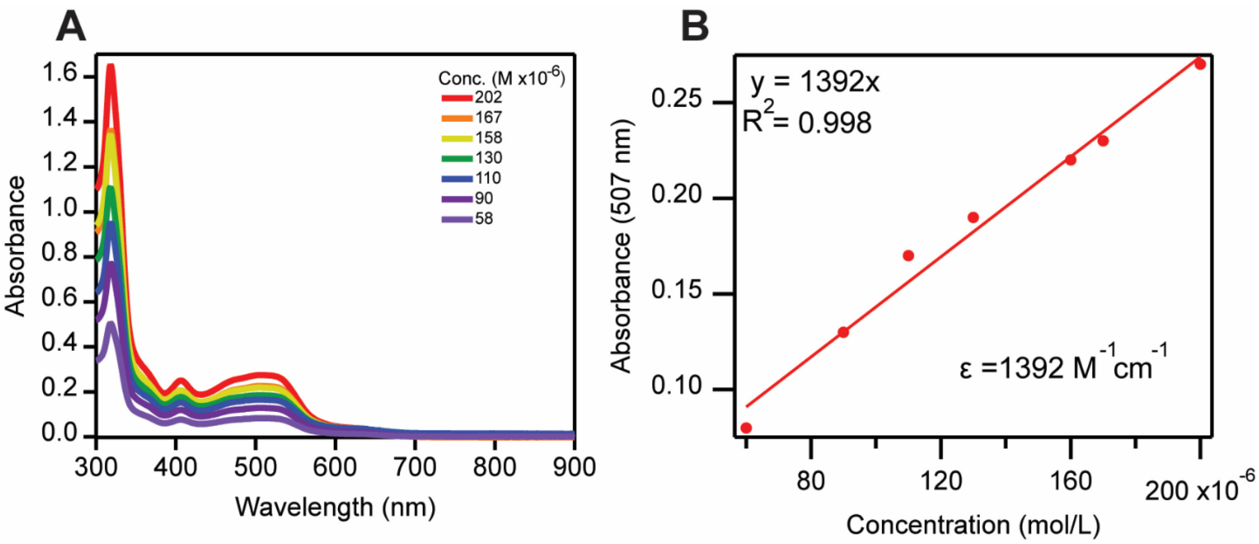

Figure S1: (A) UV-Vis absorbance spectra of $[Fe(tpy)(Mebim\text{-}py)(NCCH_3)][PF_6]_2$ in CH_3CN recorded at concentrations ranging from 58 to 220 x 10^{-6} M. (B) Beer-Lambert plot of absorption at 507 nm against sample concentration. The molar absorption coefficient at λ= 507 nm is 1,392 M^{-1} cm^{-1}.

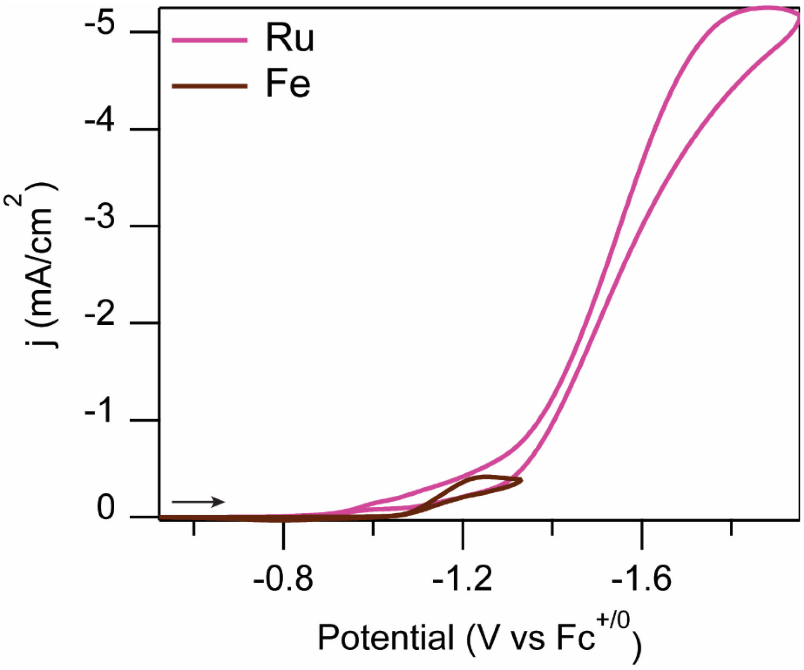

Figure S2: Cyclic voltammograms of 1 mM [Fe(tpy)(Mebim-py)($NCCH_3$)][PF_6]$_2$ solution (brown) and of 1 mM [Ru(tpy)(Mebim-py)($NCCH_3$)][PF_6]$_2$ (pink) at an illuminated p-Si–CH_3 photoelectrode under CO_2 atmosphere. Voltammograms recorded at 0.1 V s^{-1} in 100 mM [NBu_4][PF_6] 95:5 CH_3CN:H_2O solution with a Pt mesh counter electrode (in a separate compartment), and a 10 mM Ag/$AgNO_3$ reference electrode. Light source was a warm white light LED with irradiance of 339 mW cm^{-2}. Arrow indicates starting point and scan direction.

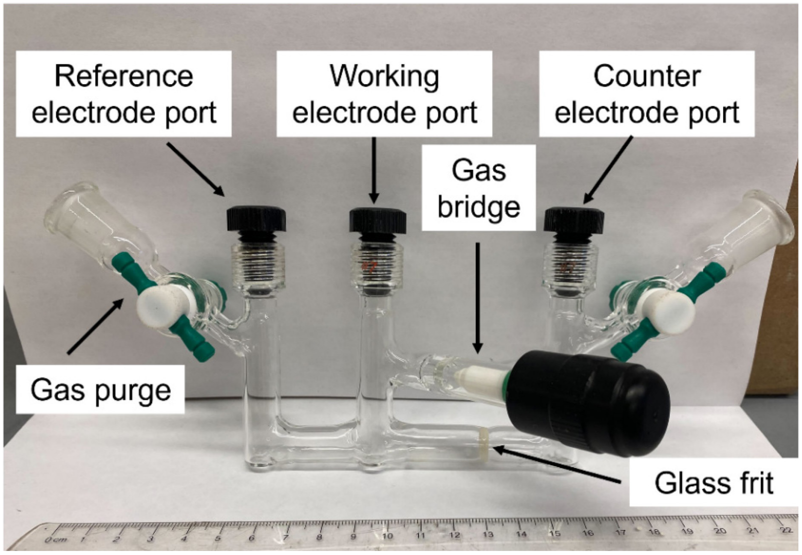

Figure S3: Photograph of the H-cell used for photoelectrolysis experiments. Electrodes were sealed in the bushings with o-rings or with septa for an airtight seal. The glass side arms allowed for a gas inlet for sparging, and the stopcocks were closed to maintain a CO_2 atmosphere.

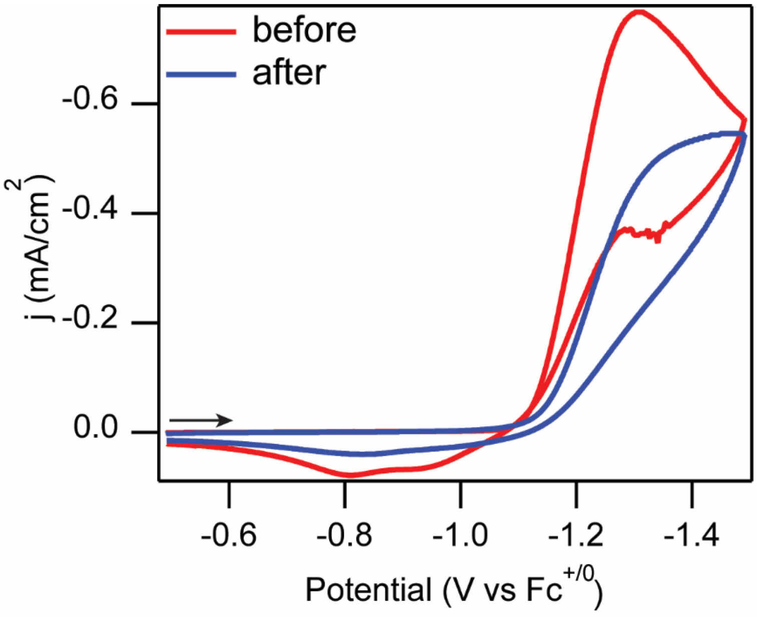

Figure S4: Cyclic voltammograms of 1 mM [Fe(tpy)(Mebim-py)(NCCH$_3$)]$^{2+}$ catalyst solution under CO_2 atmosphere before (red) and after (blue) photoelectrolysis at E_{app} = –1.31 V vs Fc$^{+/0}$ over 3600 s. Voltammograms and CPPE recorded in divided electrolysis cell at 100 mV s^{-1} in 100 mM [NBu$_4$][PF$_6$] 95:5 CH_3CN:H_2O solution with a p-Si–CH$_3$ photoelectrode, Pt mesh counter electrode (in separate compartment), and 10 mM Ag/AgNO$_3$ reference electrode with AM1.5G illumination (100 mW cm^{-2}). Arrow indicates starting point and scan direction.

Product Analysis

Henry's Law was applied to calculate the amount of CO and H_2 dissolved in the working compartment solution. The total amount in moles of product (n_p) was calculated as follows:

$$n_p = \frac{(ppm_{gas}\, V_{hs}) * 10^{-6}}{RT}\left(1 + H_s\, V_{liq}\, \frac{RT}{V_{hs}}\right) \qquad \textbf{Eq S1}$$

where ppm_{gas} is the concentration of product in the headspace sample as determined by GC (µL/L), V_{hs} is the headspace volume (L), R is the ideal gas constant (0.0821 L mol^{-1} K^{-1}), T is temperature (298 K), H_s is Henry's Law solubility constant in CH_3CN (3.343 x 10^{-3} M atm^{-1} for H_2 or 8.3 x 10^{-3} M atm^{-1} for CO)[4,5] and V_{liq} is the working compartment solution volume (L).

Faradaic Efficiency for Cathode Half-Reaction. Total moles of product n_p allowed for the calculation of Faradaic Efficiency (FE):

$$FE = \frac{n_p * n_{e^-}}{mol\ e^-} \text{ and } mol\ e^- = \frac{Q}{\mathcal{F}} \qquad \textbf{Eq S2}$$

where n_{e^-} is the number of electrons in the reduction process (2 for CO_2 reduction to CO), Q is the charge passed in C, and $\mathcal{F}$ is Faraday's constant (96485 C mol^{-1}).

Table S1: Bulk CO_2 Photoelectrolysis Metrics for 1 h CPPE in a three electrode cell.

Potential (V vs $Fc^{+/0}$)	Illumination	Charge (C)	CO produced (mol)	H_2 produced (mol)[1]	FE_{CO} (%)	FE_{H2} (%)
–1.31	AM1.5G, 100 mW/cm^2	0.374	1.01 x 10$^{–6}$	n.d.	52	--
–1.31	AM1.5G, 100 mW/cm^2	0.408	8.25 x 10$^{–7}$	n.d.	39	--
–1.31	AM1.5G, 100 mW/cm^2	0.541	1.18 x 10$^{–6}$	7.49 x 10$^{–8}$	42	3
				Average	44±6	1±1
–1.10	WWLED, 339 mW/cm^2	0.144	3.46 x 10$^{–7}$	n.d.	46	--
–1.19	WWLED, 339 mW/cm^2	0.151	3.94 x 10$^{–7}$	n.d.	50	--
–1.20	WWLED, 339 mW/cm^2	0.204	3.48 x 10$^{–7}$	n.d.	33	--
–1.70	WWLED, 339 mW/cm^2	0.362	9.84 x 10$^{–7}$	n.d.	52	--

[1]n.d. not detected

Table S2: Energy Storage CO_2 Photoelectrolysis Two-Electrode Cell Results at E_{cell} = –1.2 V.

	j (mA/cm^2)	Charge (C)	CO produced (mol)	FE for CO (%)
1	–0.183	0.266	4.32 x 10^{-7}	31
2	–0.192	0.275	1.06 x 10^{-6}	71
3	–0.202	0.288	9.71 x 10^{-7}	65
Average	–0.192	0.276	8.21 x 10^{-7}	56 ± 18

Faradaic Efficiency for Anode Half-Reaction. The change in OCP of the Fc/Fc^+ solution (ΔOCP = OCP_2–OCP_1) after a one-hour CPPE was measured to calculate the Faradaic efficiency of ferrocene oxidation at the anode that accompanies the results of Table S2. We first solved the Nernst equation for the ratio [Fc^+]/[Fc] in the product solution.

$$E = \frac{RT}{nF} \ln\left(\frac{[Fc^+]}{[Fc]}\right) + E^{0\prime} \qquad \textbf{Eq S3}$$

where E was the final OCP (V), $E^{0\prime}$ was treated as the initial OCP value.

The difference between the final and initial ratios of $[Fc^+]/[Fc]$ (Δ ($[Fc^+]/[Fc]$)) reported on the quantity of Fc^+ produced. Since the stoichiometry of the full cell reaction (Eq. 2 in main text) is $1CO_2$: 2Fc, the moles of Fc^+ were divided by a factor of 2 to calculate the Faradaic Efficiency for ferrocene oxidation (**Table S3**).

Table S3: Quantification of Faradaic Efficiency for Ferrocene Oxidation

Trial	Charge (C)	ΔOCP (mV)	Δ ($[Fc^+]/[Fc]$)	Fc^+ produced (mol)	FE_{Fc+} (%)
1	0.266	4.4	0.232	5.11×10^{-4}	93
2	0.275	7.0	0.307	5.53×10^{-4}	97
3	0.288	7.3	0.325	5.84×10^{-4}	98
Average					96

References

1 S. Gonell, J. Lloret-Fillol and A. J. M. Miller, *ACS Catal.*, 2021, **11**, 615–626.
2 G. P. Bein, M. A. Stewart, E. A. Assaf, S. J. Tereniak, R. N. Sampaio, A. J. M. Miller and J. L. Dempsey, *ACS Energy Lett.*, 2024, 1777–1785.
3 A. Bansal, X. Li, I. Lauermann, N. S. Lewis, S. I. Yi and W. H. Weinberg, *J. Am. Chem. Soc.*, 1996, **118**, 7225–7226.
4 E. Brunner, *J. Chem. Eng. Data*, 1985, **30**, 269–273.
5 E. Fujita, C. Creutz, N. Sutin and D. J. Szalda, *J. Am. Chem. Soc.*, 1991, **113**, 343–353.
